# Intestinal Microbiota and Microbial Metabolites Are Changed in a Pig Model Fed a High-Fat/Low-Fiber or a Low-Fat/High-Fiber Diet

**DOI:** 10.1371/journal.pone.0154329

**Published:** 2016-04-21

**Authors:** Sonja N. Heinritz, Eva Weiss, Meike Eklund, Tobias Aumiller, Sandrine Louis, Andreas Rings, Sabine Messner, Amélia Camarinha-Silva, Jana Seifert, Stephan C. Bischoff, Rainer Mosenthin

**Affiliations:** 1 Institute of Animal Science, University of Hohenheim, Stuttgart, Germany; 2 Department of Nutritional Medicine, University of Hohenheim, Stuttgart, Germany; Wageningen University, NETHERLANDS

## Abstract

The intestinal microbiota and its metabolites appear to be an important factor for gastrointestinal function and health. However, research is still needed to further elaborate potential relationships between nutrition, gut microbiota and host’s health by means of a suitable animal model. The present study examined the effect of two different diets on microbial composition and activity by using the pig as a model for humans. Eight pigs were equally allotted to two treatments, either fed a low-fat/high-fiber (LF), or a high-fat/low-fiber (HF) diet for 7 weeks. Feces were sampled at day 7 of every experimental week. Diet effects on fecal microbiota were assessed using quantitative real-time PCR, DNA fingerprinting and metaproteomics. Furthermore, fecal short-chain fatty acid (SCFA) profiles and ammonia concentrations were determined. Gene copy numbers of lactobacilli, bifidobacteria (*P*<0.001) and *Faecalibacterium prausnitzii* (*P*<0.05) were higher in the LF pigs, while *Enterobacteriaceae* were more abundant in the HF pigs (*P*<0.001). Higher numbers of proteins affiliated to *Enterobacteriaceae* were also present in the HF samples. Proteins for polysaccharide breakdown did almost exclusively originate from *Prevotellaceae*. Total and individual fecal SCFA concentrations were higher for pigs of the LF treatment (*P*<0.05), whereas fecal ammonia concentrations did not differ between treatments (*P*>0.05). Results provide evidence that beginning from the start of the experiment, the LF diet stimulated beneficial bacteria and SCFA production, especially butyrate (*P*<0.05), while the HF diet fostered those bacterial groups which have been associated with a negative impact on health conditions. These findings correspond to results in humans and might strengthen the hypothesis that the response of the porcine gut microbiota to a specific dietary modulation is in support of using the pig as suitable animal model for humans to assess diet-gut-microbiota interactions.

Data are available via ProteomeXchange with identifier PXD003447.

## Introduction

The importance of the intestinal microbiota for gastrointestinal function and health has been shown in many studies with human subjects, but also with model animals including rodents and pigs [1]. Diet composition reflects the substrates available for the intestinal microbiota, affecting their composition and metabolic activity. Several bacteria such as species of the *Lactobacillus* or *Bifidobacterium* genera [1], or *Faecalibacterium prausnitzii* [2] have shown beneficial effects on the health of humans and animals, and may possibly be used as a biomarker of intestinal health. On the other hand, specific species of bacterial groups such as *Enterobacteriaceae* (enterotoxic *Escherichia coli*, *Shigella*) are pathogenic and are known to be detrimental for both humans and animals [3,4]. Within this regard, dietary fibers are associated with a beneficial diet having multiple effects such as regulation of the host gut bacterial community, hindgut fermentation and health, as reviewed by Anderson et al. [5]. Dietary fibers include polysaccharides and lignin, oligosaccharides and resistant starches [5]. For example, wheat bran, as a common fiber source used in human nutrition, contains about 70% carbohydrates on a moisture-free basis [6]. The beneficial effects of dietary fiber may be mediated in part by the increase of colonic fermentation and short-chain fatty acid (SCFA) production induced by the gut microbiota. Butyrate has a particularly important role for colonocyte metabolism, and a proposed role in providing protection against colon cancer and colitis [7]. On the other hand, a typical Western diet rich in animal protein, sugar, starch and fat, but low in fiber [4] has been associated with overweight and obesity, diabetes and atherosclerosis [8]. A relationship between the frequency of certain bacterial groups and the development of obesity has been suggested both for humans and rodents [9,10].

However, few studies have been directed so far on the impact of nutrition, especially using high-fat diets, on human gut microbiota or production of intestinal SCFA in prospective trials (e.g. [11]). The use of rodent models has some advantages, such as low costs in breeding, feeding and handling, but there are also limitations such as a high mortality rate and several physiological and metabolic differences compared with humans. For example, rodents are originally granivore animals, with fermentation dominating in their large cecum and also practicing cecotrophy in contrast to omnivorous human. Consequently, in view of studies concerning intestinal microbiota, inconsistencies have been observed among studies using rodents and human subjects as reviewed by Heinritz et al. [1]. Moreover, recent reviews focusing on comparisons between porcine models and rodents revealed more similarities between pigs and humans in terms of gastrointestinal anatomy and physiology, pharmaceutical bioavailability and nutrient digestibility [12,13]. In this context, the pig has been identified as a potential competitor for mice to be used as a prime microbiome research model [14]. Comparable to humans, the gut microbiota of pigs mainly consists of the *Firmicutes* and *Bacteroidetes* phyla [15]. Though, there are also differences such as the *Bifidobacterium* genus, which is not constantly present in pigs’ intestine, and members of this genus, e.g. *Bifidobacterium suis* or *Bifidobacterium globosum* in the pig differ from the ones harboring the human gastrointestinal tract [16,17]. It is generally acknowledged that primate models have the advantage of close evolutionary relatedness and physiology to humans, however, there are considerably more stringent ethical restrictions in terms of experimentation and breeding/care compared with mice or pigs [14]. Altogether, these arguments are in support of using the pig as model animal in the present study. Two different diets, referred to as high-fat/low-fiber (HF), or low-fat/high-fiber (LF), were used to assess, whether these diets will affect gut microbiota composition and formation of microbial metabolites to be used as indicators for the gut health status of the host. Simulating changes in microbial composition and activity due to dietary changes in pigs similar to those in humans could help to establish the pig as a model for the evaluation of food supplements such as pro- and prebiotics. Within this regard, mimicking two different types of diets used in human nutrition in a long-term feeding trial with pigs represents a new approach compared to previous pig studies where e.g. standard pig diets high in fat, or genetically obese (mini-) pigs were used [18–20].

## Materials and Methods

### Ethical Approval

The research protocol was approved by the German Ethical Commission for Animal Welfare (V302/12 TE). All dietary treatments were in accordance with the guidelines issued by the German regulation for care and treatments of animals.

### Animals and housing

Eight castrated male pigs (German Landrace x Piétrain) averaging 3 months in age with an initial body weight (BW) of 27.7 kg ± 1.9 kg were obtained from the Research Station of the University of Hohenheim. Before the start of the experiment, the pigs were acclimated for 10 days to their local environment, i.e. a temperature controlled room (18–20°C) equipped with infrared heating lamps. Pigs were housed individually in metabolic crates (1.5 m × 1.0 m) permitting visual and olfactory contact between animals which had free access to water by low pressure drinking nipples. Once daily, groups of four pigs each were allowed for about 3 hours to move around in an indoor paddock to maintain social contact.

### Study design

In total, eight pigs were equally and randomly allotted to two treatment groups. The first treatment received a high-fat/low-fiber diet (HF), and the second one was fed a low-fat/high-fiber diet (LF). Both diets were formulated to meet or to exceed the National Research Council [21] nutrient recommendations for pigs from 25 to 50 kg of BW. The LF diet used in the present study contained 216.8 g NDF/kg DM which is about 45% above NDF levels used in standard diets for grower pigs [22]. On the other hand, standard diets usually contain about 30 g fat/kg DM [23], whereas the fat content of the HF diet was substantially higher amounting to 249 g fat/kg DM. Ingredient composition and nutrient contents of the diets are shown in Table 1. The whole study lasted 7 weeks. Before the start of the experiment, i.e. during the acclimation period of 10 days, pigs received a commercial standard diet based on wheat and barley containing 169.6 g crude protein/kg DM and 13.23 MJ ME/kg DM. Thereafter, pigs were fed the experimental diets for a total of 7 weeks. Animals’ BW was recorded weekly to adjust their daily feed allowances to the assigned feeding level. Daily feed allowance was 3.5 and 4.9% (as fed) of pigs’ average BW for the HF and LF treatment, respectively, to account for differences in gross energy (GE) content between experimental diets (Table 1). As a result, daily calorie intake was the same for all pigs. Pigs were fed two equal meals in mash form twice daily (at 0730 and 1530). Fresh feces of every pig were sampled during feeding of the standard diet (called ‘base’), and at day 7 of every experimental week in the morning immediately after defecation. Feces were emptied into plastic tubing and kept on ice until transferring them within 10 min to a freezer to be stored at -80°C until analyses. At the end of the experiment, all pigs were taken to a slaughterhouse (butchery Egerhof, Eningen, Germany), and they were processed according to a routine slaughterhouse procedure. After slaughter, pigs’ empty carcass weights as well as intestine weights, both full and empty, were measured.

**Table 1:** Ingredient composition, nutrient and energy content of the HF and LF diets.

Ingredient, g/kg	HF	LF
Wheat	184.9	477.3
Wheat flour^a^	200.0	
Wheat bran^b^	50.0	350.0
Casein^c^	152.0	120.5
Sunflower margarine^d^	70.0	
Sweet cream butter^e^	150.0	
Soy oil	30.0	15.0
Fructose^f^	50.0	
Dextrose^g^	50.0	
Cellulose^h^	30.0	10.0
Vitamin and mineral premix^i^	17.0	13.4
Potassium chloride	1.4	
Monocalciumphosphate	5.4	
Sodium chloride		0.2
Calcium carbonate	4.3	8.5
TIO_2_	5.0	5.0
Analyzed nutrient content		
Dry matter (DM), g/kg	890.3	895.7
Crude protein, g/kg DM	210.2	244.3
Crude fat, g/kg DM	248.6	41.3
Neutral detergent fiber, g/kg DM	66.3	216.8
Gross energy, MJ/kg DM	23.3	19.2

HF, high-fat/low-fiber; LF, low-fat/high-fiber.

^a^ Siegle GmbH, Ditzingen, Germany.

^b^ BayWa AG, (Nürtingen), Germany.

^c^ Meggle, Wasserburg, Germany.

^d^ REWE Markt, Köln, Germany.

^e^ Milchwerke Schwaben (Weideglück), Neu Ulm, Germany.

^f^ Ferdinand Kreutzer Sabamühle, Nürnberg, Germany.

^g^ Roquette, Frankfurt, Germany.

^h^ Rettenmaier & Soehne, Rosenberg, Germany; from wood.

^i^ Deutsche Vilomix Tierernährung, Neuenkirchen-Vörden, Germany; provided the following quantities of minerals and vitamins per kg HF diet: 4.3 g calcium, 0.9 g phosphor, 0.9 g sodium, 0.2 g magnesium, 6800 I.E. vitamin A, 1020 I.E. vitamin D3, 42.5 mg vitamin E, 0.85 mg vitamin B1, 2.6 mg vitamin B2, 2.1 mg vitamin B6, 17 mcg vitamin B12, 1.7 mg vitamin K3(MNB), 10.6 mg niacin, 6.4 mg Ca-pantothenate, 0.4 mg folacin, 127.5 mg choline chloride, 68.0 mg iron, 8.5 mg copper, 45.4 mg manganese, 56.8 mg zinc-oxide, 1.1 mg iodine, 0.2 mg selenium, 0.1 mg cobalt. For the LF diet: 3.3 g calcium, 0.7 g phosphor, 0.7 g sodium, 0.1 g magnesium, 5360 I.E. vitamin A, 804 I.E. vitamin D3, 33.5 mg vitamin E, 0.67 mg vitamin B1, 2.1 mg vitamin B2, 1.7 mg vitamin B6, 13 mcg vitamin B12, 1.3 mg vitamin K3 (MNB), 8.4 mg niacin, 5.0 mg Ca-pantothenate, 0.3 mg folacin, 100.5 mg choline chloride, 53.6 mg iron, 6.7 mg copper, 35.8 mg manganese, 44.8 mg zinc-oxide, 0.9 mg iodine, 0.2 mg selenium, 0.1 mg cobalt

### Chemical and physical analyses

Determination of DM, crude ash, crude protein and NDF of the assay diets was performed according to official standard methods [24]. Content of GE in the diets and the fat components butter, margarine and soy oil was measured by means of a bomb calorimeter (IKA calorimeter, C200, IKA^®^-Werke GmbH & Co. KG, Staufen, Germany).

### DNA extraction

Genomic DNA of samples of each pig (n = 4 per treatment) for the baseline and each experimental week was extracted as described recently by Weiss et al. [25] using a combination of the protocol according to Yu & Morrison [26] and the QIAamp^®^ DNA Stool Mini Kit (Qiagen, Hilden, Germany). Quantity and quality of extracted DNA was determined using a ND-UV-Vis Spectrophotometer (NanoDrop Technologies, San Francisco, CA, US).

### Fingerprinting of bacterial DNA

For the fingerprinting of bacterial DNA, the same DNA extracts as for the quantitative real-time PCR were used. The 16S rRNA was amplified using PCR with universal primers (16S 27F: 50-AGA GTT TGA TCM TGG CTC AG-30; 16S 1492R: 50-TAC GGY TAC CTT GTT ACG ACT T-30) [27]. Polymerase chain reaction was performed by using DreamTaq DNA polymerase (EP0702 Thermo Fisher Scientific Biosciences GmbH, St. Leon-Rot, Germany). The amplification reactions were carried out in a thermocycler (Bio-Rad Laboratories, Munich, Germany) with an initial hold step (95°C for 10 min), 35 cycles of a three-step PCR (94°C for 30 s, 55°C for 30 s, 72°C for 1 min) and a final hold step (72°C for 10 min). Then, PCR products (10 μl) were digested with 10U of either Alu I, HpyF3 I (Dde I) or Rsa I (Thermo Fisher Scientific Biosciences GmbH, St. Leon-Rot, Germany) at 37°C for 1.5 h and subsequently inactivated during incubation at 65°C (Alu I, HpyF3 I (Dde I)) or at 80°C (Rsa I) for 20 min according to manufacturer’s instructions. The length of the DNA fragments was determined with a chip-based microfluidic capillary electrophoresis (Caliper LabChip GX, HT DNA 5K LabChip Kit; PerkinElmer Inc., Boston, USA) according to the manufacturer’s instructions. Sample-similarity matrix was generated using the Bray–Curtis coefficient [28], and the sample profiles, obtained with chip-based microfluidic capillary electrophoresis, were explored by ordination using non-metric multidimensional scaling (nMDS) [20]. Analysis of similarity (ANOSIM) (999 permutations) was used to calculate significant differences between LF and HF treatments. Treatment means were considered to be significant at *P*<0.05 [29]. The average plotting position of samples grouped by time point was calculated and re-ordinated using principal coordinate analysis (PCoA) [29,30].

### Quantitative real-time PCR

Quantitative real-time PCR was performed using previously published primer sets (S1 Table). All primers were obtained from biomers.net GmbH (Ulm, Germany). The quantification of total bacteria, *Roseburia* spp., *Lactobacillus* spp., *Bifidobacterium* spp., *Clostridium* Cluster XIVab, *Clostridium leptum* subgroup, the *Bacteroides-Prevotella-Porphyromonas* (*Bacteroides* group), *Enterobacteriaceae*, *Faecalibacterium prausnitzii*, *Enterococcus* spp. and *Prevotella* genus was performed using the CFX Connect^™^ Real-Time System (Bio-Rad Laboratories GmbH, Munich, Germany) associated with the Bio-Rad CFX Manager^™^ Software 3.1 (Bio-Rad Laboratories GmbH, Munich, Germany). Polymerase chain reaction amplification was carried out in 20 μl reaction mixture containing 10 μl KAPA SYBR FAST (PEQLAB Biotechnology GmbH, Erlangen, Germany) and 1 μl template DNA. Corresponding amounts of every primer (10 pmol/μl) and BioScience-Grade nuclease-free and autoclaved water were added to achieve respective primer concentrations in the reaction mixture (see S1 Table). Standard curves for every primer were designed using serial dilutions of the purified and quantified PCR products generated by standard PCR and genomic DNA from pig feces as previously described [25]. Quantity of purified PCR amplification products was determined using Qubit^®^ 2.0 Fluorometer (Invitrogen). Amplification conditions were 95°C for 3 min for initial denaturation, followed by 40 cycles of denaturation at 95°C for 5 s, primer annealing for 20 s (annealing temperatures: S1 Table) and if necessary, extension at 72°C for 20 s, and stepwise increase of the temperature from 65 to 95°C to obtain melting curve data. Quantification was performed in duplicate, and the mean values were calculated. Results were reported as log_10_ 16S rRNA gene copies/g fresh matter (FM).

### Protein extraction and mass spectrometric analysis

Pigs’ fecal samples were pooled per treatment at week 1 and 7 as qPCR data showed only marginal deviations in the phylogenetic composition between individual animals. Thus, metaproteomic data based on pooled fecal material appear to be suitable to display a representative picture of the respective active communities. In addition, feces samples of two pigs per treatment were collected and pooled after the conclusion of the acclimation period. Approximately 0.3 g of these fecal samples were used for protein extraction according to the Histodenz-based protocol of Haange et al. [31]. Protein extracts were purified on a short SDS-PAGE, and a single band was cut for in-gel peptide digestion using trypsin as described by Jehmlich et al. [32]. De-salted peptides were used for LC-MS/MS measurements performed on an EasyLC nano-HPLC (Proxeon Biosystems) coupled to an LTQ Orbitrap Elite (Thermo). Separations of the peptide mixtures were done on a 15 cm fused silica emitter of 75 μm inner diameter (Proxeon Biosystems), in-house packed with reversed-phase ReproSil-Pur C18-AQ 3 μm resin (Dr. Maisch GmbH). Peptides were injected with solvent A (0.5% acetic acid) at a flow rate of 500 nl/min and separated at 200 nL/min. Separation was performed using a linear 216 min gradient of 5–33% solvent B (80% ACN in 0.5% acetic acid). Every sample was run as a technical triplicate. LTQ Orbitrap Elite was operated in the positive ion mode. Precursor ions were acquired in the mass range from m/z 300 to 2000 in the Orbitrap mass analyzer at a resolution of 120,000 followed by MS/MS spectra acquisition. The 15 most intense precursor ions from the full scan were sequentially fragmented. High resolution HCD MS/MS spectra were acquired with a resolution of 15,000 and a target value of 40,000. The normalized collision energy was set to 35, activation time to 0.1 ms and the first mass to 120 Th. Fragmented masses were excluded for 60 s after MS/MS. The target values were 1E6 charges for the MS scans in the Orbitrap and 5000 charges for the MS/MS scans with a maximum fill time of 100 ms and 150 ms respectively. Raw MS and MS/MS data were processed by Thermo Proteome Discoverer software (v.1.4.1.14), Mascot (v. 2.4) and the NCBInr databases (NCBI_20140824.fasta, no. of sequences: 48094830) for bacteria and *Sus scrofa*. Oxidation of methionine was set as variable modification and carbamidomethylation of cysteine as fixed modification. Precursor ion tolerance was defined at 10 ppm and fragment ion tolerance to 0.02 Da. Furthermore, all peaks besides the top 12 peaks per 100 Da in each MS/MS were removed to de-noise spectra before identification. Using Thermo’s Proteome Discoverer, the default filter was set to one peptide per protein and a Mascot Significance threshold of 0.05. Protein grouping was enabled with a minimum peptide confidence of medium and a delta Cn better than 0.15. Strict maximum parsimony principle was applied with a false discovery rate of 1% based on the number of hits using a decoy database [33]. For assigning functional categories to protein groups, the Cluster of Orthologous Groups (COG) annotation system from WebMGA with an e-value cutoff of 10^−3^ and exclusively considering the best hit was used [34]. Venn diagrams were computed with jvenn [35]. The mass spectrometry proteomics data have been deposited to the ProteomeXchange Consortium [36] via the PRIDE partner repository with the dataset identifier PXD003447.

### Analyses of microbial metabolites

Fecal short-chain fatty acid concentrations were measured by gas chromatography (HP 6890 Plus GCSystem) using 4-methyl-iso-valerianic acid as the internal standard. Samples (n = 4 per treatment) were prepared along the principles described by Zijlstra et al. [37] for feces. Ammonia concentration was determined with the aid of a gas-sensitive electrode, combined with a digital voltmeter (Mettler-Toledo): 4 x 2.5 g of homogenized sample were diluted (1:7) with distilled water and centrifuged for 20 min (4,750 × *g*). The supernatant fluids were pooled, diluted (1:10) and 50 mL of the solution mixed with 0.5 mL of 10 *M* NaOH. The ammonia released was measured as different voltage in mV.

### Statistical analyses of quantitative real-time PCR and microbial metabolite data

Homogeneity of variances and normal distribution of the data were confirmed by analysis of the residuals, using the UNIVARIATE procedure of the Statistical Analysis System (SAS, SAS Institute, Inc., Cary, NC). Initially, the following linear model for selecting a repeated correlation structure was considered: yijk = μ + βj + δi + (βδ)ij + εijk; where yijk = jth measurement on kth animal in ith treatment, μ = general term (fixed), βj = effect of jth week (fixed), δi = effect of treatment (fixed), (βδ)ij = effect of jth week × ith treatment (random), εijk = error associated with yijk (random). The errors εijk of repeated measurements on the same subject (animal within treatment) are assumed to be serially correlated. Different serial correlation structures were fitted by the REML method as implemented in the MIXED procedure of SAS, and the best structure according to the Akaike Information Criterion was selected. The following models were considered for eijk: independent animal effect (compound symmetry), AR(1) and AR(1) + animal effect. Using the selected correlation structure, the data were subjected to a mixed model analysis using the MIXED procedure of SAS. Furthermore, multiple comparisons among experimental weeks within treatments were performed using a t-test, with degrees of freedom determined by the Kenward-Roger method [38]. This test was performed only when a preliminary simple F-test [39] showed differences at *P*≤0.05 (SLICE = treatment option in MIXED). The same test was used for multiple comparisons among treatments within experimental weeks (SLICE = week option in MIXED). Significant differences between treatments were represented by different superscript letters using the algorithm for letter-based representation of all pair wise comparisons according to Piepho [40]. Correlations between bacterial numbers and SCFA concentrations were calculated and subjected to Pearson’s correlation method using the CORR procedure of SAS.

## Results

### General observations

The pigs remained healthy throughout the experiment, and readily consumed their feed allowances. The analyzed chemical composition of the assay diets is shown in Table 1. Pigs’ BW increased with time, yet not differing between treatments. The BW at slaughtering day was 72.5 kg for the HF and 77.1 kg for the LF treatment, however, empty carcass weight was similar for both treatments, amounting to 57.6 and 57.2 kg for HF and LF treatment, respectively. On average, the weight of the full and empty intestine was 7.0 kg for the HF and 9.9 kg for the LF treatment, and 2.6 kg for the HF, and 3.3 kg for the LF treatment, respectively. The weight of the full and empty intestine relative to pigs’ BW was 9.8% for the HF and 12.9% for the LF treatment, and 3.7% for the HF and 4.3% for the LF treatment, respectively.

### Fingerprinting of bacterial DNA

**Fig 1:**
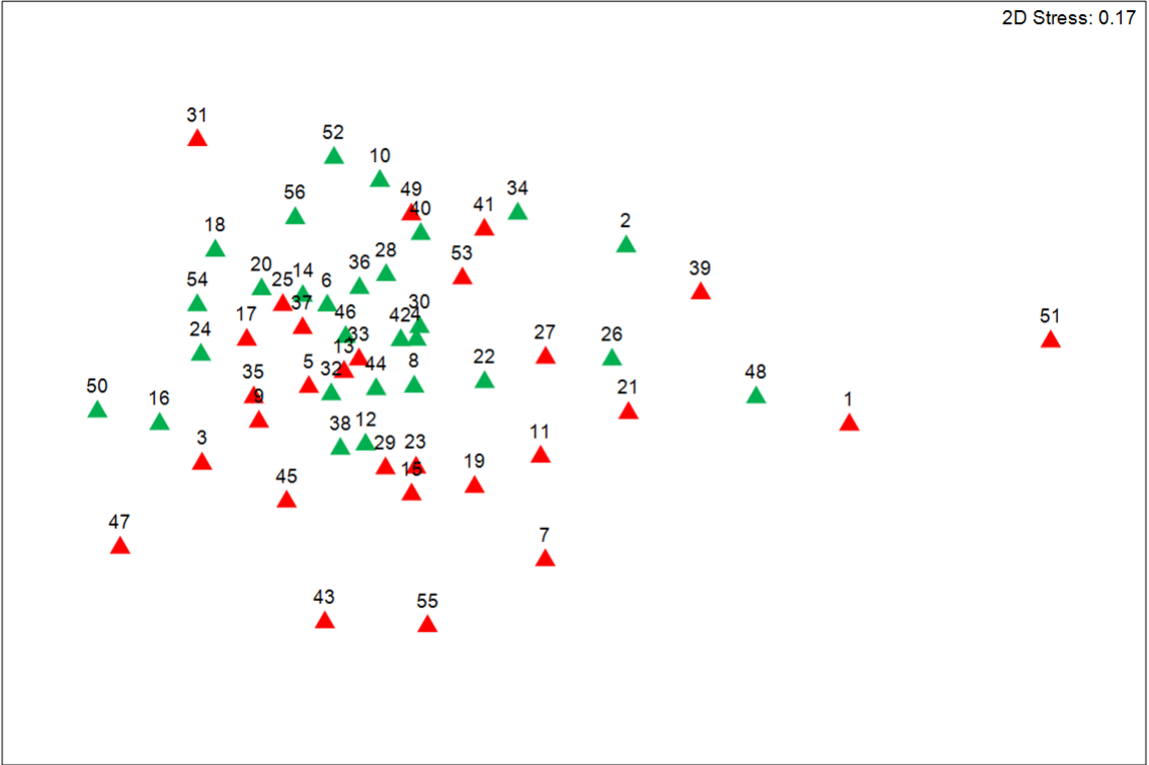
Non-metric multidimensional scaling (nMDS) plot comparing the fingerprint of pigs (n = 4 per treatment) fed the HF and LF diet for seven weeks using chip-based capillary electrophoresis data. HF, high-fat/low-fiber (▲); LF, low-fat/high-fiber (▲). Data were standardized (%) but untransformed prior to the use of the Bray–Curtis similarity algorithm. A 2D stress value of 0.17 indicates that there is no real prospect of misinterpretation. See S2 Table for identification of sample numbers.

**Fig 2:**
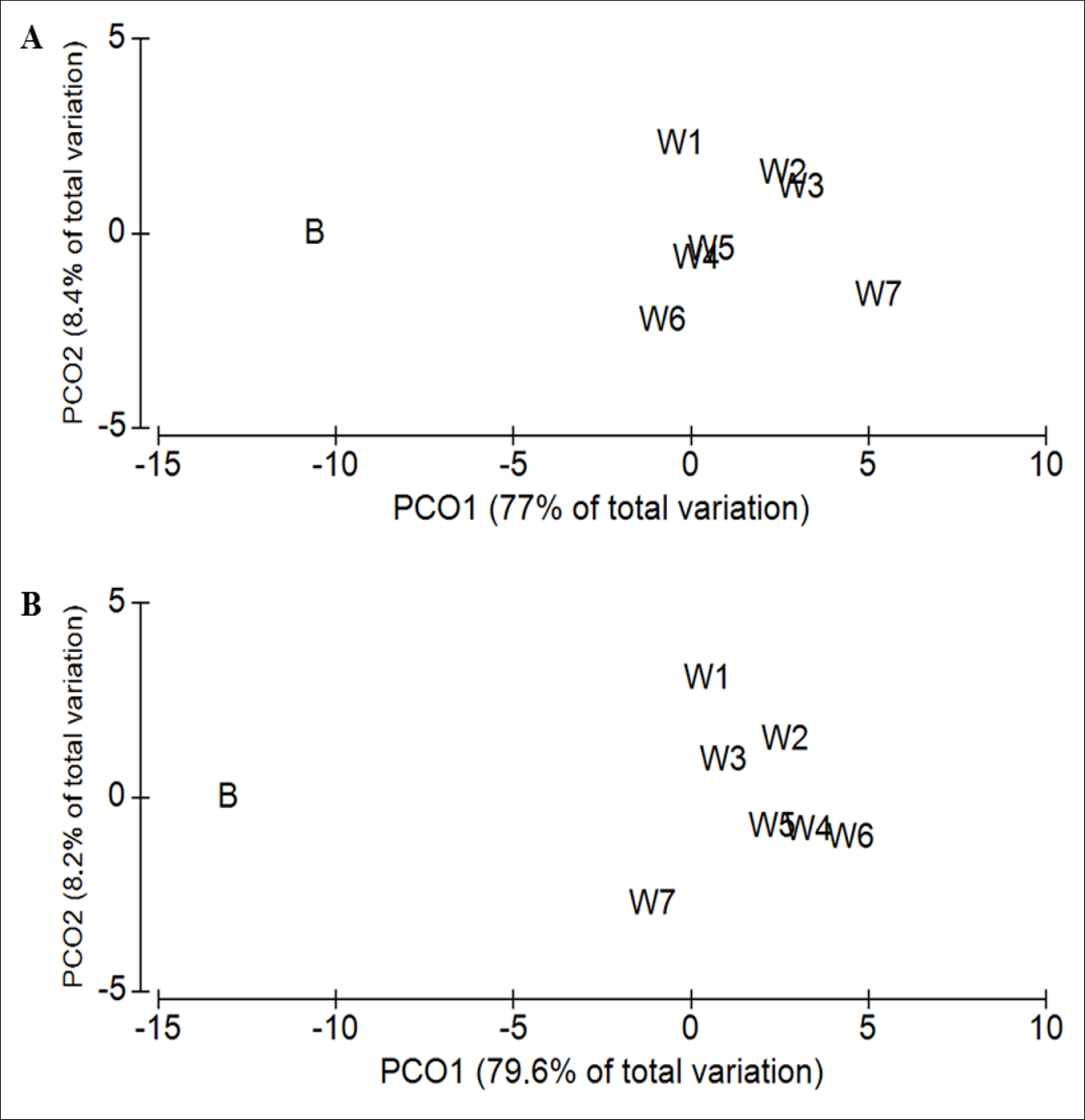
Principal coordinate analysis (PCoA) ordination of the fingerprint data of pigs (n = 4 per treatment) using chip-based capillary electrophoresis from base to week 7 for (A) LF, and (B) HF pigs, where the centroids, representing the average plotting position of the four pigs sampled at each week, are ordinated. HF, low-fat/high-fiber; LF, low-fat/high-fiber; B, base samples; W, week. Data were standardized (%). The Bray Curtis similarity algorithm was used to measure similarity between centroids. PCO1 and PCO2 account for (A) 85.4% and (B) 87.8% of the total original variation between weeks.

When analyzing digested 16S rRNA samples using microfluidic capillary electrophoresis, significant differences were observed in the nMDS plot (R = 0.153, *P* = 0.001, Fig 1) between the HF and LF treatment in all experimental periods. A clear separation of the base samples compared to observations from the experimental weeks is shown by a PCO analysis for both dietary treatments with PCO1 and PCO2 accounting for 85.4% (LF) and 87.8% (HF) of the total original variation between weeks (Fig 2).

### Effect of diet composition on the total fecal microbiota

**Fig 3:**
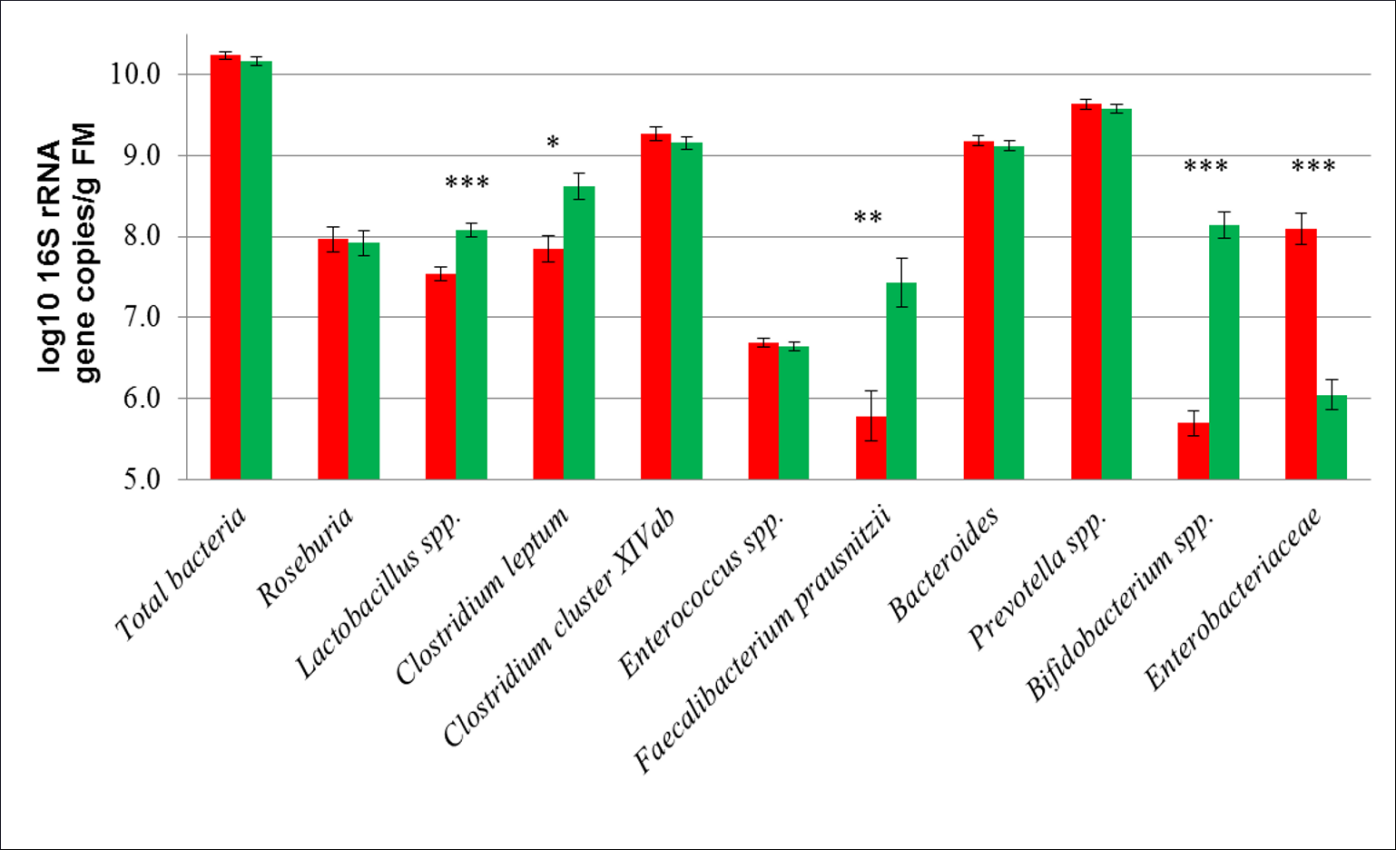
Mean values of bacterial numbers in feces of pigs (n = 4 per treatment) fed the HF and LF diet for seven weeks. HF, high-fat/low-fiber (red bars); LF, low-fat/high-fiber (green bars); FM, fresh matter. Values represent least squares means ± SEM. *P*<0.05*, *P*<0.01**, *P*<0.001***.

**Fig 4:**
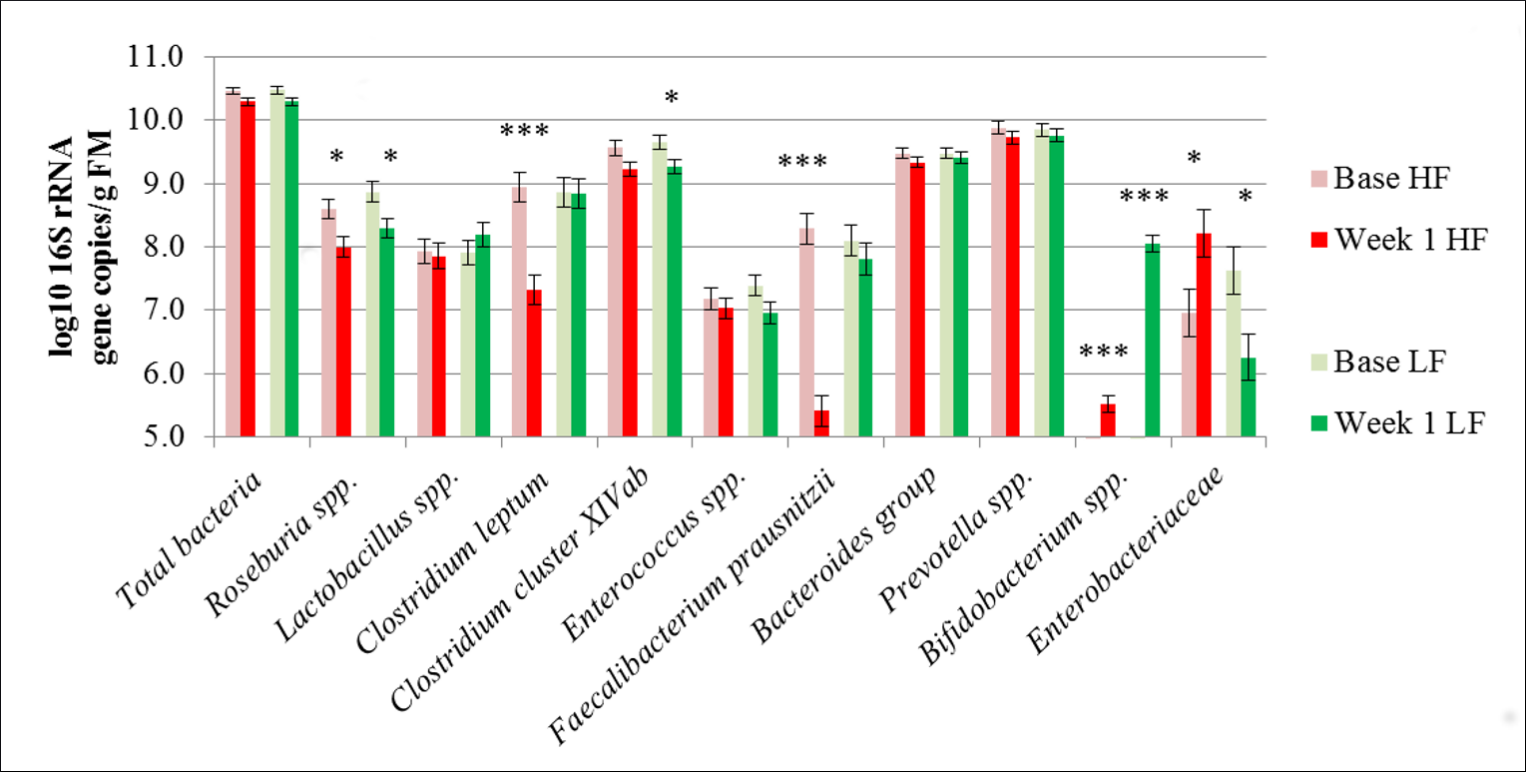
Mean values of bacterial numbers in feces of pigs (n = 4 per treatment) at the base status compared to week 1 of the experiment, HF and LF. HF, high-fat/low-fiber; LF, low- fat/high-fiber; FM, fresh matter. Values represent least squares means ± SEM. *P*<0.05*, *P*<0.01**, *P*<0.001***.

**Fig 5:**
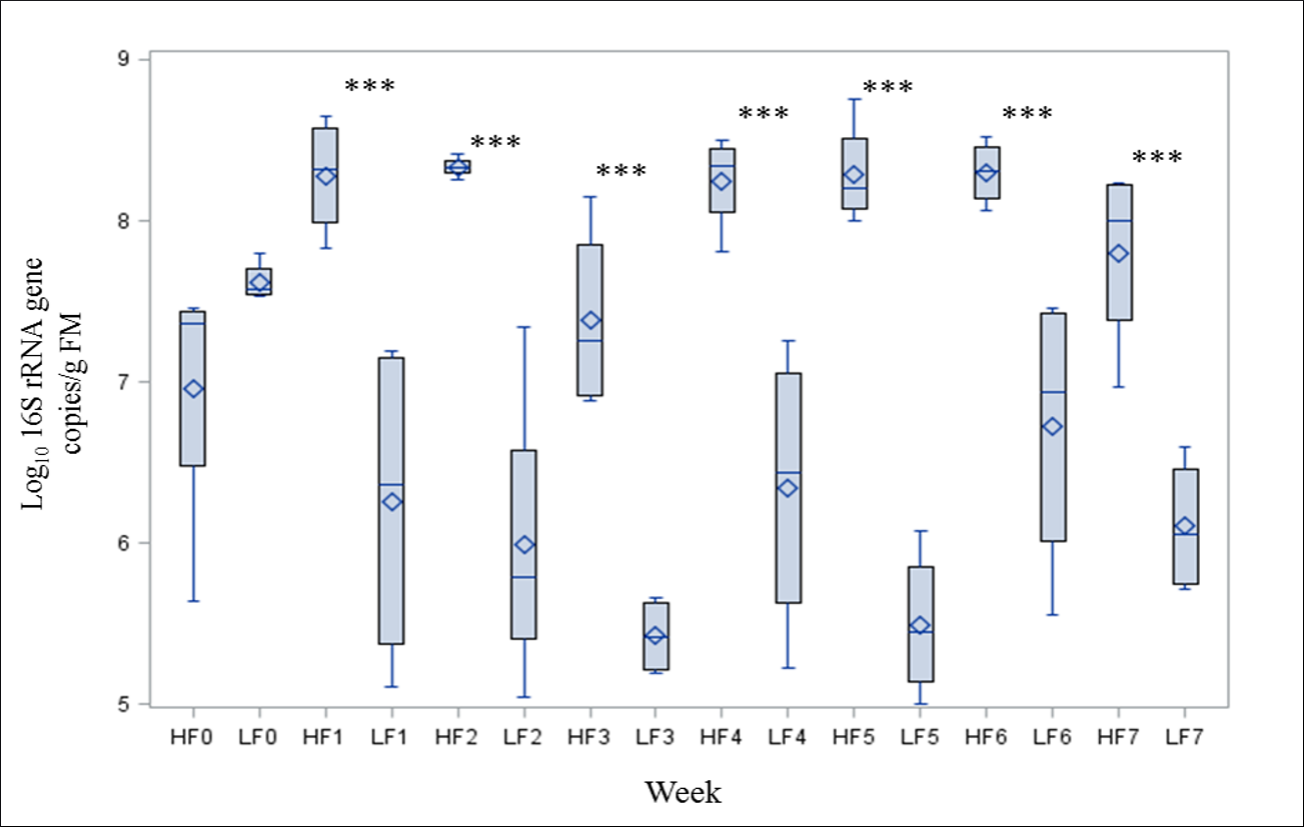
Box-and-Whisker plots of enterobacteria numbers in feces of pigs (n = 4 per treatment). HF, high- fat/low-fiber; LF, low-fat/high-fiber; FM, fresh matter. For HF and LF from base status (0) to week 7. In all the plots, upper and lower bounds of the box denote the 75th and 25th percentiles, the symbol in the box interior represents the group mean, the horizontal line in the box interior represents the group median and the vertical lines (whiskers) issuing from the box extend to the group minimum and the maximum values. *P*<0.001***.

The influence of dietary treatments on fecal bacterial gene copy numbers comprising experimental weeks 1 to 7 is shown in Fig 3 as mean values. *Enterobacteriaceae* amounted to 8.1 log_10_ 16S rRNA gene copies/g FM in the HF treatment compared to lower numbers of 6.0 log_10_ 16S rRNA gene copies/g FM in the LF treatment (*P*<0.001). Mean values of *Bifidobacterium* spp. averaged 5.7 log_10_ 16S rRNA gene copies/g FM in the HF pigs, and a more than two decimal powers higher value of 8.1 log_10_ 16S rRNA gene copies/g FM in the LF treatment (*P*<0.001). Furthermore, *Lactobacillus* spp., *C*. *leptum* and *F*. *prausnitzii* showed greater 16S rRNA gene copy numbers for the pigs of the LF treatment (*P*<0.001, *P* = 0.016 and *P* = 0.009, respectively). Interactions between treatment and experimental week (Table 2) were observed for the *Bacteroides* group, as gene copy numbers of these bacterial group decreased during the last 3 weeks, both for the HF and LF treatment (*P*<0.001). *Roseburia* 16S rRNA gene copy numbers decreased for the pigs of the LF treatment (*P* = 0.014) towards the end of the experiment with lower numbers in week 6 and 7 compared to week 1 and 2. Further interactions between experimental week and the LF treatment were also found for numbers of *Enterobacteriaceae* (*P* = 0.012), and in the HF treatment for *C*. *leptum* (*P* = 0.004), *Bifidobacterium* spp. (*P* = 0.033) and *Enterococcus spp*. (*P* = 0.032). Comparing the base status of the pigs with the first experimental week (Fig 4, mean values), *C*. *leptum* and *F*. *prausnitzii* decreased for the pigs of the HF treatment (*P*<0.001). *Roseburia* spp. decreased in both the HF and the LF treatment (*P* = 0.018 and *P* = 0.023, respectively), while *Clostridium* cluster XIVab only decreased in the LF treatment (*P* = 0.040). *Enterobacteriaceae* increased for the HF treatment (*P* = 0.048) and decreased for the pigs of the LF treatment (*P* = 0.024), and their numbers remained greater in the HF than in the LF treatment despite individual variations between pigs during the experiment (week 1–7) (Fig 5). Base samples of pigs assigned to the HF treatment showed lower gene copy numbers of enterobacteria than the ones assigned to the LF diet, yet these differences were not statistically significant (Fig 5). *Bifidobacterium* spp. were not detectable in both treatments before the start of the experiment.

**Table 2:** Development of bacterial numbers in feces of pigs (n = 4 per treatment) over time (log10 16S ribosomal RNA gene copies/g FM).

				Experimental Week						Pooled SEM	*P*-Values	
			1	2	3	4	5	6	7		Diet	Week*Diet (HF or LF)
	Total bacteria	HF	10.3	10.3	10.3	10.3	10.2	10.1	10.2	0.08	0.322	0.361
		LF	10.3	10.2	10.2	10.2	10.2	10.0	10.1			0.194
*Firmicutes*	*Roseburia* spp.	HF	8.0	8.2	7.9	7.8	8.2	7.9	7.9	0.22	0.847	0.574
		LF	8.3^z^	8.2^yz^	7.9^wz^	7.8^wxy^	8.0^xz^	7.5^w^	7.7^wx^			0.014
	*Lactobacillus* spp.	HF	7.9	7.2^a^	8.0	7.5	7.4^a^	7.3	7.4	0.22	<0.001	0.145
		LF	8.2	8.2^b^	8.2	8.2	8.1^b^	7.7	7.9			0.609
	*Clostridium leptum*	HF	7.3^awx^	6.9^aw^	8.3^y^	7.7^x^	8.3^y^	8.0^xy^	8.4^y^	0.31	0.016	0.004
		LF	8.8^b^	8.7^b^	8.6	8.6	8.8	8.4	8.4			0.817
	*Clostridium* XIVab	HF	9.2	9.3	9.4	9.2	9.3	9.2	9.2	0.15	0.360	0.925
		LF	9.3	9.3	9.3	9.1	9.0	9.0	9.2			0.406
	*Enterococcus* spp.	HF	7.0^x^	6.7^wx^	6.4^w^	6.6^wx^	6.8^x^	6.8^x^	6.4^w^	0.14	0.637	0.032
		LF	7.0	6.8	6.6	6.5	6.6	6.5	6.5			0.218
	*F*. *prausnitzii*	HF	5.4^a^	5.0^a^	6.1^a^	5.8^a^	6.3^a^	5.7	6.2	0.43	0.009	0.077
		LF	7.8^b^	7.7^b^	7.5^b^	7.4^b^	7.7^b^	7.0	7.0			0.378
*Bacteroidetes*	*Bacteroides group*	HF	9.3^w^	9.4^w^	9.4^w^	9.4^w^	9.0^x^	8.8^x^	8.9^x^	0.10	0.490	<0.001
		LF	9.4^z^	9.3^yz^	9.1^xy^	9.3^yz^	8.9^wx^	8.9^wx^	8.8^w^			<0.001
	*Prevotella* spp.	HF	9.7	9.7	9.5	9.7	9.7	9.4	9.6	0.11	0.512	0.346
		LF	9.8	9.7	9.4	9.6	9.6	9.6	9.5			0.291
*Actinobacteria*	*Bifidobacterium* spp.	HF	5.5^aw^	5.6^aw^	5.3^aw^	6.6^ax^	6.0^awx^	5.8^awx^	5.1^aw^	0.32	<0.001	0.033
		LF	8.1^b^	8.3^b^	8.2^b^	8.3^b^	8.5^b^	7.7^b^	7.9^b^			0.506
*Proteobacteria*	*Enterobacteriaceae*	HF	8.3^a^	8.3^a^	7.4^a^	8.3^a^	8.3^a^	8.3^a^	7.8^a^	0.31	<0.001	0.097
		LF	6.3^bx^	6.0^bwx^	5.4^bw^	6.3^bx^	5.5^bw^	6.7^bx^	6.1^bwx^			0.012

FM, fresh matter; HF, high-fat/low-fiber; LF, low-fat/high-fiber. With regard to the diet, data not sharing the same letter (a,b) within a column and for one parameter assessed are significantly different (*P*<0.05);With regard to the experimental weeks, data not sharing the same letter (wxyz) within a row are significantly different, for HF or LF (*P*<0.05). Values represent least squares means.

### Effect of diet composition on the active fecal microbiota

**Fig 6:**
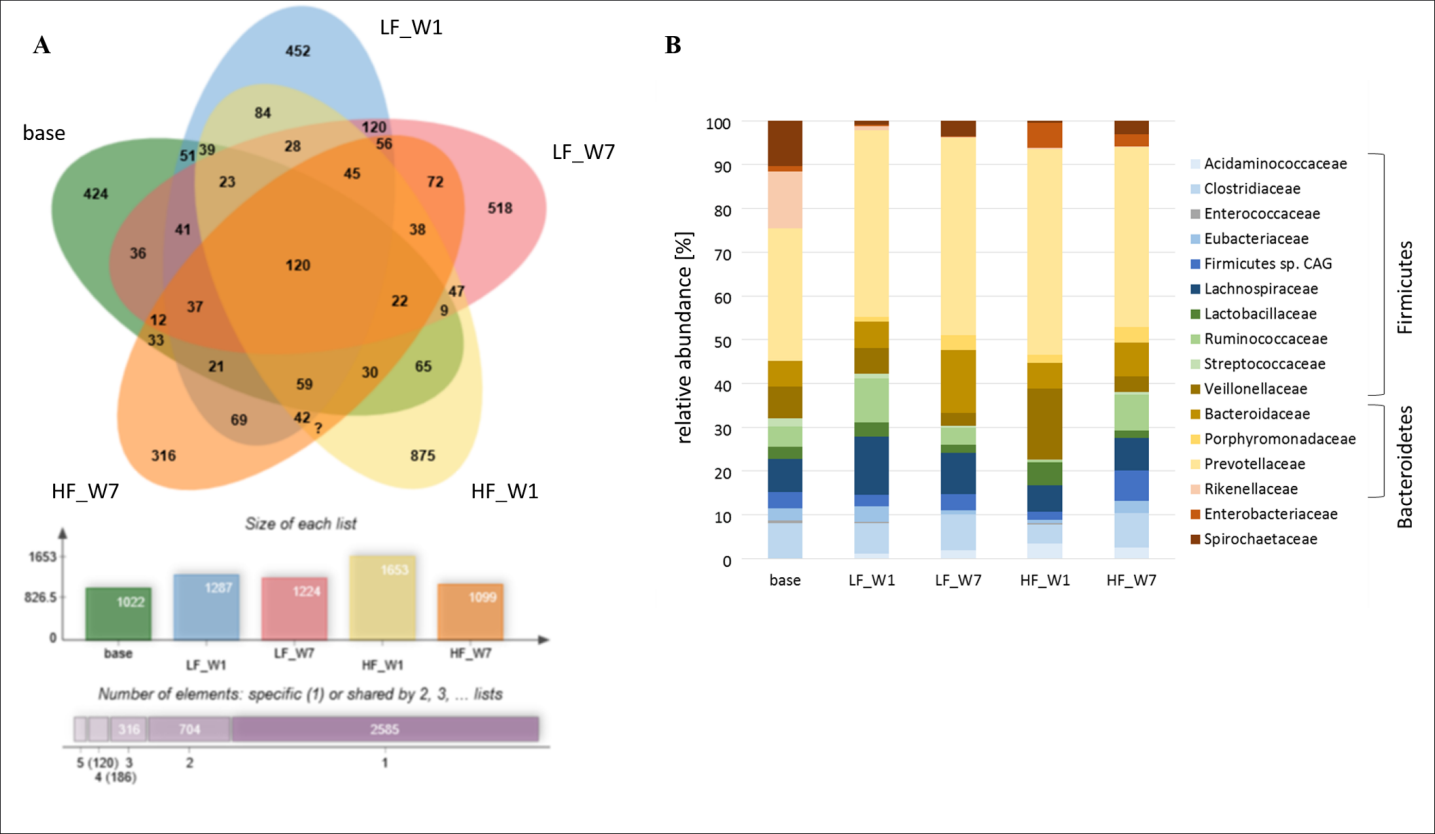
Distribution of identified peptides (A) and phylogenetic distribution of identified proteins (B) in the fecal samples (n = 1 each, measured in technical triplicates) from the base animals, HF and LF diet treated animals (W1: week 1 and W7: week7). HF, high-fat/low-fiber; LF, low-fat/high-fiber.

**Fig 7:**
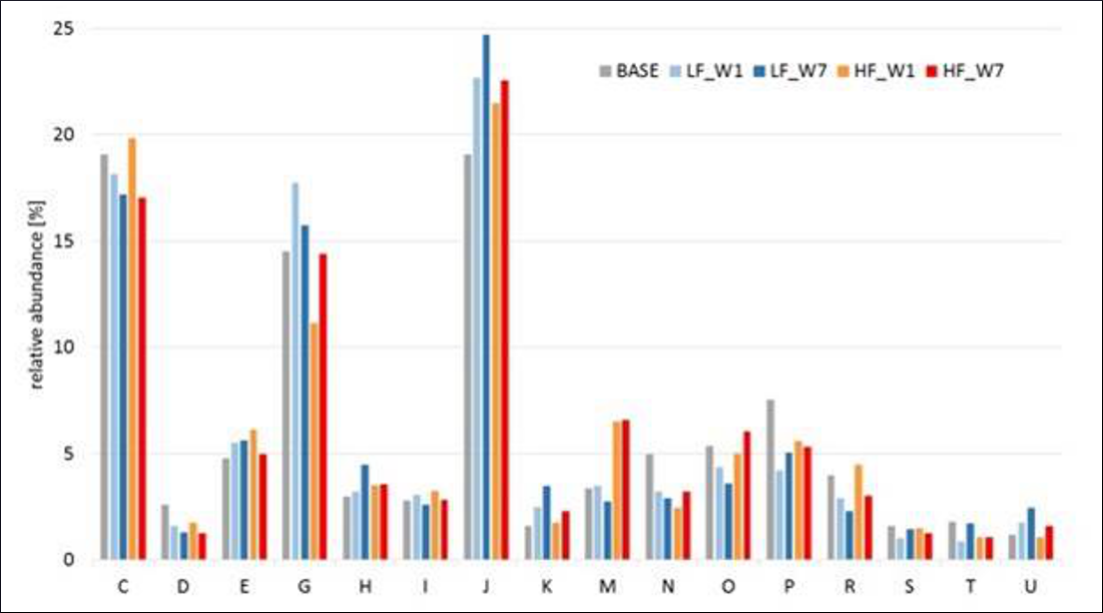
Distribution of cluster of orthologous groups (COG) classes of proteins identified in the fecal samples (n = 1 each, measured in technical triplicates) from the base animals, HF and LF diet treated animals (W1: week 1 and W7:week7). HF, high-fat/low-fiber; LF, low-fat/high-fiber; C, Energy production and conversion; D, Cell cycle control and mitosis; E, Amino Acid metabolism and transport; G, Carbohydrate metabolism and transport; H, Coenzyme metabolism; I, Lipid metabolism; J, Translation; K, Transcription; M, Cell wall/membrane/envelop biogenesis; N, Cell motility; O, Post-translational modification, protein turnover, chaperone functions; P, Inorganic ion transport and metabolism; R, General Functional Prediction only; S, Function Unknown; T, Signal Transduction; U, Intracellular trafficing and secretion.

The metaproteomic analyses included five fecal samples to assess if there exist time-dependent treatment effects on the active bacterial community. These samples comprised one base sample, and four samples were pooled from both treatments each at week 1 and 7. There were about 500 to 740 identified protein groups per sample (1020–1650 identified peptides, Fig 6) including 5–19% proteins affiliated to *Sus scrofa* (S3 Table). As shown in the Venn diagram (Fig 6A), only 120 proteins were common in all samples and each sample showed a high fraction of unique proteins. Obviously, diet shaped the protein inventory already after one week, as only 51 and 65 proteins in LF and HF samples of week 1, respectively, corresponded with proteins found in the base sample, whereas 120 proteins in LF samples of week 1 were common with LF samples in week 7. The majority of proteins belonged to 16 different families of the phyla *Bacteroidetes*, *Firmicutes*, *Gammaproteobacteria* and *Spirochaetes* (Fig 6) including *Prevotella*, *Bacteroides*, *Clostridium*, *Lachnospira*, *Alistipes*, *Ruminococcus*, *Dialister*, *Selenomonas* and *Treponema* as the main genera. Only minor differences in the phylogenetic composition between fecal samples of the base, LF and HF treatments were observed. The ratio between *Firmicutes* and *Bacteroidetes* was about 0.8 in the base fecal sample, increased to 0.95 in the sample of LF (week 1) and decreased to 0.5 at week 7. However, this ratio did hardly change in fecal samples of the HF treatment in comparison to the base samples. Proteins belonging to *Rikenellaceae* (*Alistipes* spp.) made up 13% in the base sample but decreased to 0.5% upon feeding of the experimental diets. The ratio between *Enterobacteriaceae*: *Lactobacillaceae* changed from 0.5 in the base sample to 0.13 in the two LF samples, and to 0.75 in the two HF samples. The functional annotation of the proteins using COG classes showed an almost equal distribution between all fecal samples (S4 Table). Major COG classes were C (Energy production and conversion), E (Amino acid transport and metabolism), G (Carbohydrate transport and metabolism) and J (Translation, ribosomal structure and biogenesis) (Fig 7). A decrease of proteins belonging to G was observed in the fecal sample of the HF treatment in week 1 compared to the base sample, whereas there was an increase in proteins belonging to M (Cell wall/membrane/envelope biogenesis) in samples of the HF treatment collected in week 1 and 7. Proteins from members of the *Prevotellaceae* were predominant in all samples (30–47%, Fig 6), and these bacteria seemed to be the major players in polysaccharide degradation and SCFA formation. In each samples, except for base and HF week 7, an alpha-amylase belonging to a *Prevotella* spp. was identified. In addition, in week 1 an 1,4-alpha-glucan branching enzyme was detected for LF treatment. In the HF samples, proteins related to inulin (2,6-beta-D-fructofuranosidase) and arabinoxylan (alpha-L-arabinofuranosidase, xylanase) were found. Besides one putative cellulosomal scaffoldin protein from *Ruminococcus* spp., no other carbohydrate active enzymes were identified in the samples. Proteins from the subsequent catabolic routes, like pyruvate:ferredoxin (flavodoxin) oxidoreductase and acetate kinase could also be linked to members of the *Prevotellaceae*.

### Effect of diet composition on fecal microbial metabolites

Dietary treatment affected concentrations of both total and individual SCFA in fecal samples (Table 3). During the whole experiment, pigs fed the HF diet had lower concentrations of acetate compared to the LF treatment (*P*<0.001), with average contents of 124.3 and 287.8 mmol/kg DM for the HF and LF treatment, respectively. Similarly, butyrate concentration was lower in the HF treatment (*P* = 0.018) with 38.7 mmol/kg DM compared to 61.1mmol/kg DM in the LF treatment, and propionate amounted to 72.8 mmol/kg DM in the HF treatment compared to 102.7 mmol/kg DM in the LF treatment (*P* = 0.021) (Table 3). As a result, total SCFA concentration in the HF treatment was lower during the 7 experimental weeks compared to the LF treatment (*P* = 0.002). There were no treatment effects on fecal ammonia concentrations, and there were also no interactions between treatment and experimental week (Table 3).

**Table 3:** Development of short-chain fatty acids (SCFA) and ammonia contents in feces of pigs (n = 4 per treatment) over time.

		Experimental Week							Pooled SEM	*P*-Values	
		1	2	3	4	5	6	7		Diet	Week*Diet (HF or LF)
SCFA, mmol/kg of DM											
Acetate	HF	110.5^a^	108.5^a^	134.6^a^	121.8^a^	123.0^a^	123.8^a^	147.8^a^	26.08	<0.001	0.837
	LF	285.5^bwxy^	314.2^bwy^	268.9^bwx^	305.3^bwxy^	338.1^by^	248.8^bx^	253.8^bx^			0.029
Propionate	HF	77.9	74.6	63.7^a^	75.5	76.0^a^	76.4^a^	65.9	11.69	0.021	0.924
	LF	91.6	102.8	105.7^b^	104.8	112.4^b^	111.6^b^	89.7			0.534
Butyrate	HF	39.5^a^	34.0^a^	30.2^a^	35.5^a^	43.9^a^	42.2	45.7	6.84	0.018	0.131
	LF	61.9^b^	72.4^b^	54.4^b^	61.0^b^	67.9^b^	55.5	54.9			0.148
Total^1^	HF	270.0^a^	258.1^a^	255.9^a^	270.5^a^	283.4^a^	281.8^a^	293.4^a^	42.71	0.002	0.923
	LF	481.4^b^	537.2^b^	469.5^b^	517.5^b^	569.1^b^	486.6^b^	442.5^b^			0.090
Ammonia, mmol/kg of DM	HF	12.7	10.9	9.1	11.7	13.8	13.0	13.3	1.31	0.249	0.176
	LF	14.7	12.6	10.6	12.1	14.9	14.4	11.0			0.131

DM, dry matter; HF, high-fat/low-fiber; LF, low-fat/high-fiber. With regard to the diet, data not sharing the same letter (a,b) within a column and for one parameter assessed are significantly different (*P*<0.05); With regard to experimental weeks, data not sharing the same letter (wxy) within a row are significantly different, for HF or LF (*P*<0.05). Values represent least squares means.

^1^ Includes iso-forms.

## Discussion

Both DNA fingerprint data and qPCR results revealed a considerable effect of diet composition on fecal microbial community, as gut microbiota composition differed distinctly upon feeding HF and LF diets compared to the base status during the whole experiment. For the LF treatment, greater *Bifidobacterium* spp. gene copy numbers could be determined due to the known stimulating effect of dietary fibers on saccharolytic bifidobacteria as reviewed by Slavin [41]. For example, xylooligosaccharides present in wheat bran have shown their potential to stimulate certain *Bifidobacterium* strains *in vitro* [42]. In contrast, no proteins affiliated to *Bifidobacteriaceae* were identified in any of the samples. Similarly, Loh et al. [43] could not detect *Bifidobacterium* spp. in colonic samples of growing pigs, which needs to be considered when using the pig as a model for humans, as these bacteria harbor the gastrointestinal tract of humans. In the present study, *Bifidobacterium* spp. were also not present in base samples but could be detected by means of qPCR upon feeding of the experimental diets. The reasons for this discrepancy remain unclear, but it needs to be pointed out that bifidobacteria generally constitute a smaller amount of the microbiota in pigs according to Loh et al. [43]. In comparison to other studies, fecal gene copy numbers of lactobacilli and enterobacteria of pigs of both treatments were considerably lower than those reported by Hermes et al. [44]. These authors fed high-fiber diets based on rice and barley and supplemented with wheat bran and sugar beet pulp, and used the same extraction and similar quantification methods as reported herein. Differences in age among pigs may be held responsible, at least in part, for this discrepancy, especially concerning lactobacilli [1], as in the study of Hermes et al. [44] pigs aged 8 weeks, compared to 3 months at the start of the experiment in this study. On the other hand, Pieper et al. [45] found similar gene copy numbers for total bacteria and lactobacilli in the proximal colon of pigs (50 d old) fed a corn-wheat-soybean meal-based diet, but higher numbers of *Bacteroides* and *C*. *leptum* compared to both treatments in the present study.

Concerning *Enterobacteriaceae*, metaproteomic analyses confirmed results of the real-time PCR analysis, observing greater numbers in the HF than in the LF treatment, and allowed a specific detection of proteins associated with possible pathogenic strains (*E*.*coli*, *Salmonella enterica*) in the HF samples (Fig 6). These proteins included mainly ribosomal proteins and outer membrane porins belonging to *E*. *coli* and *Salmonella enterica*, whereas no proteins assigned to the pathogenic properties of these bacteria were found. In contrast, higher concentrations of total SCFA as observed for the LF treatment, have been reported to be closely associated with a decrease in luminal pH [41], thus creating a hostile environment e.g. for some acid-sensitive bacteria strains of *E*. *coli*. Similarly, Smith et al. [46] found a decrease in the *Enterobacteriaceae* population in association with higher concentrations of total SCFA in the large intestine of finisher-pigs when feeding barley-based diets compared to oat-based diets. Accordingly, in the present study, a negative correlation was found between *Enterobacteriaceae* numbers and acetate, propionate and butyrate concentrations (S5 Table). In a human study, greater numbers of *Enterobacteriaceae* were found in feces of European children consuming a typical Western diet high in animal protein, sugar, starch, and fat and low in fiber compared to children from rural Africa living on more vegetarian diets low in fat and animal protein and rich in starch, fiber, and plant polysaccharides [4]. Furthermore, increased *Enterobacteriaceae* numbers have been detected in overweight pregnant women [47], and they were more abundant in type 2 diabetes compared to control patients [48].

It can be concluded that diet composition, BW and health status of humans are closely associated with the abundance of *Enterobacteriaceae* in fecal samples, which is in support of our observations, where this bacterial group was more prevalent in feces of pigs fed the HF diet than in the LF diet.

In the present study, acetate and butyrate concentrations in feces of pigs were positively correlated with numbers of fiber fermenting bifidobacteria, lactobacilli, *C*. *leptum* and *F*. *prausnitzii*, while numbers of *Roseburia* spp. were positively correlated with butyrate only (S5 Table). *Bifidobacterium* and *Lactobacillus* spp. are known for their beneficial effects on human health, and are therefore used as probiotic food ingredients, as reviewed by Heinritz et al. [1]. For example, they attach to enterocytes, thereby inhibiting the binding of enteric pathogens due to competitive exclusion [49], and they influence commensal microorganisms by the production of lactic acid and bacteriocins. These substances inhibit growth of pathogens and also alter the ecological balance of enteric commensals [49]. *F*. *prausnitzii* is a prominent butyrate forming bacteria often associated with members of the *C*. *leptum* group, which is predominant in the colonic microbiota of healthy humans, with changes in the abundance of *F*. *prausnitzii* having been described for several intestinal and metabolic diseases in humans [2]. In the present study, lower 16S rRNA gene copy numbers of *F*. *prausnitzii* were found along with lower butyrate concentrations in the HF treatment, which is supported by a positive correlation of *F*. *prausnitzii* and butyrate (S5 Table) and being in agreement with observations of Ramirez-Farias et al. [50] in human feces. In addition, the metaproteomic analysis detected 8 proteins of this species in the LF (week 1) sample, whereas no proteins of *F*. *prausnitzii* were detected at the same time in the HF sample. Identified proteins indicated a transport mechanism of glucose via the PTS, the oxidative decarboxylation of pyruvate and formation of acetoacetyl-CoA by an acetyl-CoA acetyltransferase, which is one of the first steps in the butyrate formation pathway. In agreement with results in human studies, the pig model has proven to be suitable to confirm the butyrate producing capacity of *F*. *prausnitzii*. As shown by qPCR data, *Prevotellaceae* were abundant in both dietary treatments, which could be confirmed by metaproteomic data. It appears that *Prevotellaceae* represents an important bacterial group for polysaccharide degradation and the formation of SCFA. All enzymes involved in polysaccharide (starch) degradation identified here were associated with this group. Recent metagenomic studies [51] confirmed the prevalence of *Prevotellaceae* in the cecum, colon and feces of pigs. Accordingly, Frese et al. [52] reported an increase in *Prevotellaceae* following introduction of a more favorable, polysaccharide containing diet after weaning due to their genetic capacity for polysaccharide degradation. Thus, higher abundance of *Prevotellaceae* could have been expected in the LF treatment due to a higher content of polysaccharides (216.8 g NDF/kg DM) compared to the HF treatment (66.3 g NDF/kg DM). It remains open, however, if differences in the composition of polysaccharides among studies can be held responsible for the discrepancy between the results of the study by Frese et al. [52] and the results of this study.

In humans, special attention has been given to bifidobacteria, lactobacilli, *C*. *leptum* and *F*. *prausnitzii* due to their inverse relationship with obesity and overweight [47,53,54], and to the ratio between *Firmicutes* and *Bacteroidetes* [1]. In general, a higher fraction of *Firmicutes* than *Bacteroidetes* was identified in several DNA-based studies using porcine feces [51,55]. In addition, there was a higher number of *Firmicutes* in fecal samples of obese humans and in intestinal samples of obese Ossabaw mini-pigs [1,19]. These findings, however, could not be confirmed by the result of this study, where protein counts of the two phyla showed diet-independent higher abundances of *Bacteroidetes* than *Firmicutes*. In addition, there was no increase of *Firmicutes* proteins in HF samples. In contrast, the LF sample obtained in week 7 showed an increased abundance of proteins affiliated to *Bacteroidaceae*, whereas proteins from *Lachnospiraceae* and *Ruminococcaceae* decreased. In the present study, lactobacilli were less abundant in HF than in LF pigs. Similarly, Pedersen et al. [19] found less lactobacilli in colon digesta of obese Ossabaw mini-pigs fed a high energy diet (42.9% fat) than in lean animals given a standard chow diet (10.5% fat). Correspondingly, diverse *Lactobacillus* spp. have been successfully applied as probiotics to treat obesity in humans [56,57], whereas in other studies with humans increased *Lactobacillus* concentrations have been associated with obesity and BW gain [58,59]. The authors of these studies emphasized, however, that genomic differences even within a single *Lactobacillus* species have to be accounted for. For example, according to Drissi et al. [59], weight protection-associated genomes encoded more bacteriocins than those associated with BW gain. In the present work, feeding a high-fiber diet resulted in higher lactobacilli numbers pointing towards beneficial effects on microbial composition such as higher bifidobacteria and *F*. *prausnitzii* numbers, although no differences in pigs’ BW were observed. Concerning bifidobacteria and *C*. *leptum*, their abundance was reduced in overweight and obese adults in a study of Schwiertz et al. [53], yet the authors hesitated to draw final conclusions on the participation of various bacterial groups in the development of obesity. Lower bifidobacteria numbers were also observed in obese children in the study of Kalliomäki et al. [54]. Taken together, the LF diet fostered bacterial groups, which have been positively recognized for showing beneficial effects on humans’ health [2,49] whereas they were present in significant lower numbers in pigs fed the HF diet.

Concerning SCFA, there is a curvilinear relationship between transit time and fecal concentrations of total and individual SCFA (especially butyrate). At intestinal transit times exceeding 50 h, butyrate is not any longer detectable in fecal samples, but used as energy source of the colonocytes [60]. Previous studies showed that SCFA are rapidly absorbed from the colonocytes with only 5% being excreted in feces, as reviewed e.g. by den Besten et al. [61]. The abundance of SCFA in feces represents the net production of SCFA above their absorption rate. In accordance with a report by De Filippo et al. [4], the results of the present study allow us to hypothesize that a diet rich in plant polysaccharides and low in sugar and fat promotes proliferation of SCFA-producing bacteria. On the other hand, there is evidence that branched-chain SCFA might be produced by fermentation of protein reaching the large intestine following ingestion of plant protein sources usually not completely digested by host enzymes. Yet, in combination with a larger amount of fermentable carbohydrates as supplied by the LF diet in this study, non-digested protein accumulating in the large intestine will stimulate bacterial proliferation, thereby enhancing SCFA production, as reviewed by Millet et al. [62].

The observed differences in concentrations between the experimental diets in the present study are in agreement with Yan et al. [20] who fed a high-fat (17.5% swine grease) corn-based diet, or a low-fat diet (5% swine grease) to pigs. The authors determined lower concentrations of acetate, propionate and butyrate in cecal samples upon feeding the high-fat compared to the low-fat treatment. No explanation was given for these observations, though this result could be ascribed to an inhibitory effect of fat on microbial fermentation as observed in ruminants [63]. Increased concentrations of SCFA in the gastrointestinal tract have been associated with reduced risk of some diseases including the irritable bowel syndrome, inflammatory bowel disease, cardiovascular disease and cancer in humans [5]. In the present study, using the pig as animal model, it could be demonstrated that the LF diet based on wheat and wheat bran and containing 216.8 g NDF/kg DM enhanced fecal butyrate concentrations, obviously due to higher non-starch polysaccharide contents compared to the HF diet containing 66.3 g NDF/kg DM. However, a recent study with mice revealed that elevated levels of butyrate upon feeding of a high-fiber diet enhanced the cell-killing capacity of *E*. *coli* O157:H7 Shiga toxin through higher colonic and renal Shiga toxin receptor levels. Furthermore, the high-fiber diet led to decreased numbers of commensal *Escherichia* spp. compared to mice fed a low-fiber diet, but resulted in higher colonization levels of *E*. *coli* O157:H7, more weight loss and greater mortality rates in mice fed on a high-fiber diet [64]. In pigs, Shiga toxins produced by *E*. *coli* cause systemic vascular damage that manifests as edema disease [65], possibly indicating a limited use of the pig as model when fed on high-fiber diets. Though, host specific variations in intestinal ecology and diet, as well as environmental factors such as age have to be considered [64]. Nevertheless, due to the assumed health promoting effects of dietary fiber, future studies should elucidate in more detail the underlying mechanisms behind the dietary fiber concept [66].

In this study, there were no differences in fecal ammonia concentrations between treatments which corresponds well with an equal abundance of proteins related to *Prevotellaceae* (ca. 45%), a family known to have the capacity to degrade proteins [67], in all samples. This is somewhat surprising as the protein content supplied by cereals in the LF treatment was higher compared to the HF treatment, where highly digestible casein dominated as protein source. Thus, one might expect higher concentrations of ammonia, a potential irritant of the intestinal mucosa [44] in the LF pigs due to higher amounts of protein possibly reaching the large intestine serving as substrate for protein degrading bacterial groups [68]. Yet, the abundance of the *Enterobacteriaceae* family, containing proteolytic bacteria, was lower in the LF pigs, probably caused by higher amounts of SCFA and lower pH [41], which might have inhibited further ammonia production.

In conclusion, the implementation of the pig as animal model which can be supplied with dietary ingredients similar to those used in human nutrition might be a promising approach. There were significant differences in the microbial composition, the abundance of several important bacterial groups and metabolites between diets. Our data suggest diet as an important factor that shapes the gut microbial community in a similar way to human, which can help to establish the pig as a model for the evaluation of food supplements such as pro- and prebiotics, or the effects of SCFA in relation to markers of cancer risk [69]. Future studies with pigs should include more detailed anthropometric parameters, determination of additional metabolites of protein degradation, and the use of advanced analytical methods such as next generation sequencing to further assess the impact of nutrition on the gut microbiota. In addition, the inter-individual variability of the microbiota composition should be considered in future studies, thus analyses of metaproteomics should be done using individual rather than pooled samples. To further improve the model described herein, human microbiota-associated pigs might have the potential to mimic the human gut microbiota more authentically, and thus may be used more frequently as an animal model in future studies [70,71].

## Supporting Information

S1 Table Oligonucleotide primers used for real-time PCR. (DOCX)

S2 Table Fig 1 labeling. (DOCX)

S3 Table Table of protein IDs for all measured samples. (XLSX)

S4 Table Table of proteins and associated COG classes in all samples. (XLSX)

S5 Table Statistical data for the correlations between bacterial numbers (log10 16S ribosomal RNA) and concentrations of short-chain fatty acids in feces of pigs over seven experimental weeks. (DOCX)
