# Supplementary material for: Intestinal Microbiota and Microbial Metabolites Are Changed in a Pig Model Fed a High-Fat/Low-Fiber or a Low-Fat/High-Fiber Diet
Source: PLoS One. 2016 Apr 21;11(4):e0154329. doi: 10.1371/journal.pone.0154329 (PMC4839692; doi:10.1371/journal.pone.0154329)
Supplement: S1 Table — (DOCX) [file pone.0154329.s001.docx]

| **Targeted bacterial group (Amplicon size)** | **Item** | **Sequence** **(5'-> 3')** | **Annealing**  **Temperature (°C)** | **Primer**  **concentration**  **(nM)** | **Reference** |
| --- | --- | --- | --- | --- | --- |
| Total bacteria (147bp) | F | GTGSTGCAYGGYYGTCGTCA | 52 | 600 | [1] |
|  | R | ACGTCRTCCMCNCCTTCCTC |  |  |  |
| *Roseburia* spp. (353bp) | F | AGGCGGTACGGCAAGTCT | 59 | 400 | [2] |
|  | R | AGTTTYATTCTTGCGAACG |  |  | [3] |
| *Bacteroides-Prevotella-Porphyromonas* (140bp) | F | GGTGTCGGCTTAAGTGCCAT | 59 | 600 | [3] |
|  | R | CGGAYGTAAGGGCCGTGC |  |  |  |
| *Lactobacillus* spp. (391bp) | F | AGAGGTAGTAACTGGCCTTTA | 59 | 200 | [4] |
|  | R | GCGGAAACCTCCCAACA |  |  |  |
| *Enterobacteriaceae* (385bp) | F | ATGGCTGTCGTCAGCTCGT | 59 | 600 | [5] |
|  | R | CCTACTTCTTTTGCAACCCACTC |  |  |  |
| *Clostridium leptum* (239bp) | F | GCACAAGCAGTGGAGT | 63,3 | 600 | [6] |
|  | R | CTTCCTCCGTTTTGTCAA |  |  |  |
| *Clostridium* cluster XIVab (150bp) | F | GCA GTG GGG AAT ATT GCA | 57 | 600 | [7] |
|  | R | CTT TGAGTTTCATTCTTGCGAA |  |  |  |
| Genus *Prevotella* (121bp) | F | CACRGTAAACGATGGATGCC | 59 | 600 | [8] |
|  | R | GGTCGGGTTGCAGACC |  |  |  |
| *Bifidobacterium* spp.(126bp) | F | CGCGTCCGGTGTGAAAG | 59 | 400 | [9] |
|  | R | CTTCCCGATATCTACACATTCCA |  |  |  |
| *Enterococcus* spp. (144bp) | F | CCCTTATTGTTAGTTGCCATCATT | 59 | 400 | [3] |
|  | R | ACTCGTTGTACTTCCCATTGT |  |  |  |
| *Faecalibacterium prausnitzii* (203bp) | F | GGAGGATTGACC CCTTCAGT | 59 | 600 | [10] |
|  | R | CTGGTCCCGAAGAAACACAT |  |  |  |

**S1 Table. Oligonucleotide primers used for real-time PCR**

Bp, base pairs; F, forward; R, reverse.

1. Fuller Z, Louis P, Mihajlovski A, Rungapamestry V, Ratcliffe B, Duncan AJ. Influence of cabbage processing methods and prebiotic manipulation of colonic microflora on glucosinolate breakdown in man. Br J Nutr. 2007;98:364–72.

2. Veiga P, Gallini CA, Beal C, Michaud M, Delaney ML, DuBois A, et al. *Bifidobacterium animalis* subsp. *lactis* fermented milk product reduces inflammation by altering a niche for colitogenic microbes. Proc Natl Acad Sci USA. 2010;107:18132-7.

3. Rinttilä T, Kassinen A, Malinen E, Krogius L, Palva A. Development of an extensive set of 16S rRNA-targeted primers for quantification of pathogenic and indigenous bacteria in fecal samples by real-time PCR. J Appl Microbiol. 2004;97:1166–77.

4. Malinen E, Kassinen A, Rinttila T, Palva A. Comparison of real-time PCR with SYBR Green I or 5 ′-nuclease assays and dot-blot hybridization with rRNA-targeted oligonucleotide probes in quantification of selected faecal bacteria. Microbiology. 2003;149:269–77.

5. Castillo M, Martín-Orúe SM, Manzanilla EG, Badiola I, Martín M, Gasa J. Quantification of total bacteria, enterobacteria and lactobacilli populations in pig digesta by real-time PCR. Vet Microbiol. 2006;114:165-70.

6. Matsuki T, Watanabe K, Fujimoto J, Takada T, Tanaka R. Use of 16S rRNA gene-targeted group-specific primers for real-time PCR analysis of predominant bacteria in human feces. Appl Environ Microbiol 2004;70:7220e8.

7. Song Y, Liu C, Finegold SM. Real-time PCR quantitation of clostridia in feces of autistic children. Appl Environ Microbiol. 2004;70:6459–65.

8. Stevenson DM, Weimer PJ. Dominance of *Prevotella* and low abundance of classical ruminal bacterial species in the bovine rumen revealed by relative quantification real-time PCR. Appl Microbiol Biotechnol. 2007;75:165–74.

9. Xiang ZT, Qi HW, Han GQ, Liu J, Huang Z, Yu B. Real-time TaqMan polymerase chain reaction to quantify the effects of different sources of dietary starch on Bifidobacterium in the intestinal tract of piglets. Afr J Biotechnol. 2011;10:5059–67.

10. Balamurugan R, Janardhan HP, George S, Raghava MV, Muliyil J, Ramakrishna BS. Molecular studies of fecal anaerobic commensal bacteria in

acute diarrhea in children. J Pediatr Gastroenterol Nutr. 2008;46:514-19.
