# Supplementary material for: Intestinal Microbiota and Microbial Metabolites Are Changed in a Pig Model Fed a High-Fat/Low-Fiber or a Low-Fat/High-Fiber Diet
Source: PLoS One. 2016 Apr 21;11(4):e0154329. doi: 10.1371/journal.pone.0154329 (PMC4839692; doi:10.1371/journal.pone.0154329)
Supplement: S2 Table — (DOCX) [file pone.0154329.s002.docx]

**S2 Table. Figure 1 labeling**

| Sample nr | Animal | Week |
| --- | --- | --- |
| 1 | S1 | W1 |
| 2 | S2 | W1 |
| 3 | S3 | W1 |
| 4 | S4 | W1 |
| 5 | S5 | W1 |
| 6 | S6 | W1 |
| 7 | S7 | W1 |
| 8 | S8 | W1 |
| 9 | S1 | W2 |
| 10 | S2 | W2 |
| 11 | S3 | W2 |
| 12 | S4 | W2 |
| 13 | S5 | W2 |
| 14 | S6 | W2 |
| 15 | S7 | W2 |
| 16 | S8 | W2 |
| 17 | S1 | W3 |
| 18 | S2 | W3 |
| 19 | S3 | W3 |
| 20 | S4 | W3 |
| 21 | S5 | W3 |
| 22 | S6 | W3 |
| 23 | S7 | W3 |
| 24 | S8 | W3 |
| 25 | S1 | W4 |
| 26 | S2 | W4 |
| 27 | S3 | W4 |
| 28 | S4 | W4 |
| 29 | S5 | W4 |
| 30 | S6 | W4 |
| 31 | S7 | W4 |
| 32 | S8 | W4 |
| 33 | S1 | W5 |
| 34 | S2 | W5 |
| 35 | S3 | W5 |
| 36 | S4 | W5 |
| 37 | S5 | W5 |
| 38 | S6 | W5 |
| 39 | S7 | W5 |
| 40 | S8 | W5 |
| 41 | S1 | W6 |
| 42 | S2 | W6 |
| 43 | S3 | W6 |
| 44 | S4 | W6 |
| 45 | S5 | W6 |
| 46 | S6 | W6 |
| 47 | S7 | W6 |
| 48 | S8 | W6 |
| 49 | S1 | W7 |
| 50 | S2 | W7 |
| 51 | S3 | W7 |
| 52 | S4 | W7 |
| 53 | S5 | W7 |
| 54 | S6 | W7 |
| 55 | S7 | W7 |
| 56 | S8 | W7 |

nr, number; S, sample; W, week
