# Supplementary material for: Intestinal Microbiota and Microbial Metabolites Are Changed in a Pig Model Fed a High-Fat/Low-Fiber or a Low-Fat/High-Fiber Diet
Source: PLoS One. 2016 Apr 21;11(4):e0154329. doi: 10.1371/journal.pone.0154329 (PMC4839692; doi:10.1371/journal.pone.0154329)
Supplement: S5 Table — (DOCX) [file pone.0154329.s005.docx]

**S5 Table. Statistical data for the correlations between bacterial numbers (log_10_ 16S ribosomal RNA) and concentrations of short-chain fatty acids in feces of pigs over seven experimental weeks**^a^

|  | Acetate | | Propionate | | Butyrate | |
| --- | --- | --- | --- | --- | --- | --- |
|  | *P* | *R* | *P* | *R* | *P* | *R* |
| Total bacteria | NS |  | NS |  | NS |  |
| *Roseburia* spp. | NS |  | NS |  | 0.011 | 0.344 |
| *Lactobacillus* spp. | <0.001 | 0.585 | 0.013 | 0.334 | <0.001 | 0.442 |
| *Clostridium leptum* | <0.001 | 0.653 | NS |  | <0.001 | 0.639 |
| *Clostridium* XIV ab | NS |  | NS |  | NS |  |
| *Enterococcus* spp. | NS |  | NS |  | NS |  |
| *F. prausnitzii* | <0.001 | 0.751 | 0.003 | 0.386 | <0.001 | 0.498 |
| *Bacteroides* group | NS |  | NS |  | NS |  |
| *Prevotella* spp. | NS |  | NS |  | NS |  |
| *Bifidobacterium* spp. | <0.001 | 0.823 | <0.001 | 0.551 | <0.001 | 0.623 |
| *Enterobacteriaceae* | <0.001 | -0.762 | 0.005 | -0.547 | <0.001 | -0.567 |

^a^ Results are presented as *P* values of <0.05 (significant) or >0.05 (NS, not significant) and *R* values for the significant correlations.
